# Appearance and re-appearance of zoonotic disease during the pandemic period: long-term monitoring and analysis of zoonosis is crucial to confirm the animal origin of SARS-CoV-2 and monkeypox virus

**DOI:** 10.1080/01652176.2022.2086718

**Published:** 2022-06-16

**Authors:** Chiranjib Chakraborty, Manojit Bhattacharya, Shyam Sundar Nandi, Ranjan K. Mohapatra, Kuldeep Dhama, Govindasamy Agoramoorthy

**Affiliations:** a Department of Biotechnology, School of Life Science and Biotechnology, Adamas University, Kolkata, West Bengal, India;; b Department of Zoology, Fakir Mohan University, Balasore, Odisha, India;; c ICMR-National Institute of Virology, (Mumbai Unit), Indian Council of Medical Research, Haffkine Institute Compound, Mumbai, India;; d Department of Chemistry, Government College of Engineering, Keonjhar, Odisha, India;; e Division of Pathology, ICAR-Indian Veterinary Research Institute, Bareilly, Uttar Pradesh, India;; f College of Pharmacy and Health Care, Tajen University, Pingtung, Taiwan

**Keywords:** SARS-CoV-2, monkeypox virus, natural reservoirs, intermediate hosts, bats, monkey

In response to the ongoing COVID-19 pandemic, biologists are working hard to screen many species of wild animals to logically pinpoint the exact origin of the virus. Now, researchers are more alert after the second consequence of zoonotic viral diseases during the pandemic period. Previously, an editorial by Wong et al. appeared in Zoological Research that suggested tracking the origin of SARS-CoV-2. It was an important scientific question (Wong et al. 2020). Recent monkeypox spread in more than 12 non-African countries has again raised the same question to screen many species of wild animals to identify the particular origin of the monkeypox virus rationally (Graham 2022, Velavan and Meyer 2022, Yang 2022). Monkeypox is now a global threat during the COVID-19 pandemic period and the global health community is on alert (Graham 2022, Rudan 2022, Yang 2022). So the emerging and reemerging zoonotic diseases have aggravated the same question to pinpoint the exact origin of the viruses, the host range, and natural reservoir (Figure 1).

**Figure 1.**
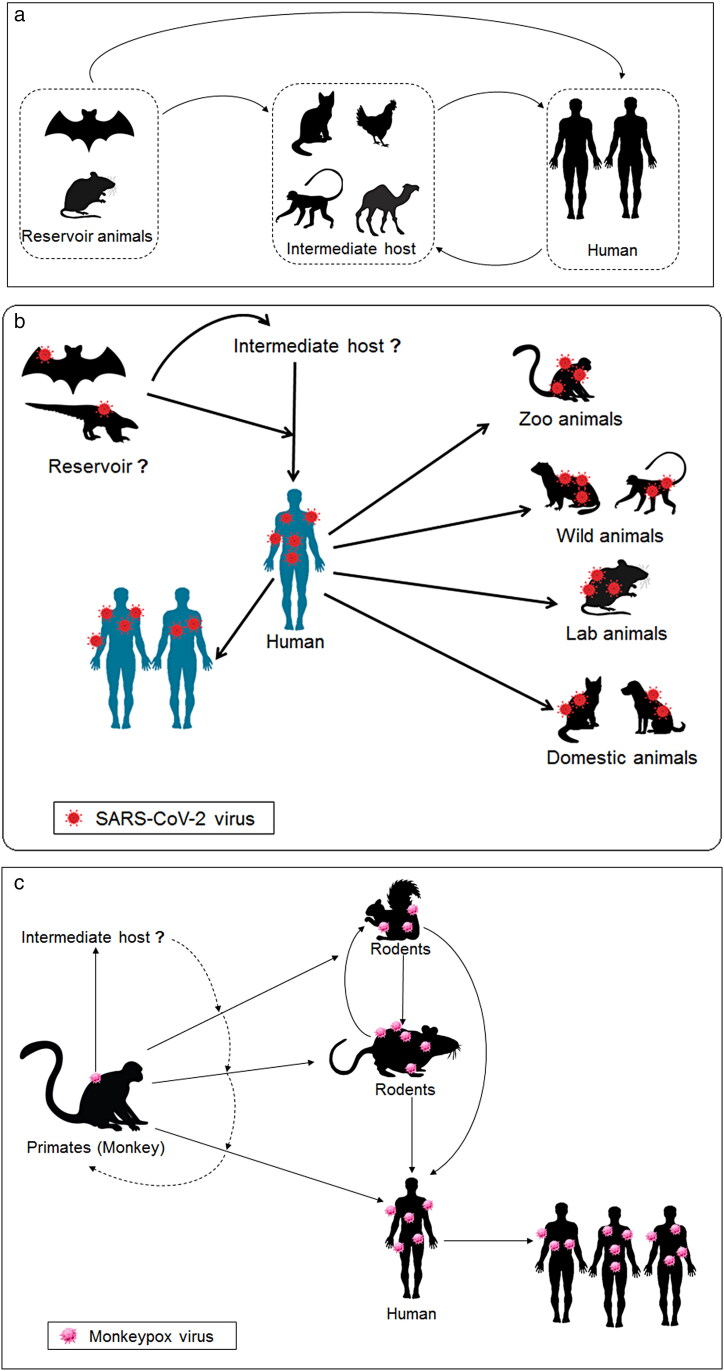
A schematic diagram of the zoonosis and the virus spillover events of two zoonotic viruses (SARS-CoV-2 and monkeypox virus). The spillover event illustrates how the virus jumps from the natural reservoir to intermediate hosts and from intermediate hosts to human or other hosts (a). A schematic diagram illustrates general concepts of infectious viruses and their spillover event. The spillover event demonstrates how the virus jumps from natural reservoirs to intermediate hosts to humans (b). A schematic diagram illustrates general concepts of zoonosis of the SARS-CoV-2 viruses and its spillover event from the natural reservoir to intermediate hosts. It also demonstrates how the virus jumps from intermediate hosts to humans (c). A schematic diagram illustrates general concepts of the monkeypox virus's zoonosis and its spillover event from the natural reservoir to intermediate hosts. It also describes how the virus jumps from intermediate hosts to humans.

It has been observed that several infectious diseases have emerged from time to time. The causes and epidemics of these diseases influenced both the global human and animal health. Most of the emerging infectious diseases are zoonotic diseases of animal origin (Judson and Rabinowitz 2021). Several zoonotic viral infections have been noted, which are Ebola virus disease, Avian influenza, Hantavirus, Chikungunya, hand, foot, and mouth disease, Crimean-Congo hemorrhagic fever, Japanese encephalitis, Dengue, Rift Valley fever, Nipah virus, Novel human coronavirus, Rabies, Viral hepatitis, etc. Among those viruses, we know from previous reports about their reservoir host/vector (Table 1). However, in most cases such as SARS-CoV-2 and monkeypox virus, the main reservoir(s) are still unknown. Understanding the zoonotic reservoirs is essential at this point, and it will help prevent and control future infectious diseases with zoonotic nature such as SARS-CoV-2 and monkeypox virus outbreaks.

**Table 1.** Different zoonotic viral pathogens, animal host/hosts, and their year of epidemics.

Sl. No.	Name of zoonotic viral pathogen	Probable animal host	Year of epidemic	Reference
1.	Ebola and Marburg	Bats, wild birds	2018	(Cunningham et al. 2017)
2.	Nipah, Rhabdovirus	Bats, primates	2015	(Kreuder Johnson et al. 2015)
3.	Hepatitis E virus	Wild boars	2019	(Zecchin et al. 2019)
4.	Zika virus	Mosquitoes	2015	(Ioos et al. 2014)
5.	Arbovirus	Wild birds	2001	(Pereira et al. 2001)
6.	Nipah, West Nile	Chimpanzees and bats	2001	(Daszak et al. 2001)
7.	Anger virus	Bats	2015	(Núñez et al. 2012)
8.	Avian influenza H7N9/HPAIVs	Wild birds	2016	(Reperant et al. 2016)
9.	Influenza A, Corona	Bats	2017	(Hosseini et al. 2017)
10.	H5N1, H5N2, Ebola, rabies	Monkeys, African buffalo, wild ducks	2014	(Kock 2014)
11.	MERS-CoV	Camels	2012	(Gastanaduy 2013)
12.	Coronavirus, SARS	Bats	2013	(Shi 2013)
13.	SARS-CoV	Bats, pangolins	2002	(Xiao et al. 2003)
14.	Chikungunya virus	Mosquitoes	2004	(Laras et al. 2005)
15.	Parvovirus, Hantavirus	Pigs	2016	(Benavides-Arias and Soler-Tovar 2015)
16.	Rabies virus, Leptospirosis	Wild and domestic dogs	2013	(Martinez et al. 2013)
17.	SARS-CoV-2	Likely bats, pangolins, dogs	2020	(Voskarides 2022, Wu et al. 2020, Zhang et al. 2020)
18.	Lassa virus	Rats	2018	(Ehichioya et al. 2019)
19.	Dengue virus	Mosquitoes	2010	(Schwartz and Albert 2010)
20.	Ebola virus	Bats	2017	(Cunningham et al. 2017)
21.	Monkeypox virus	Monkey	2007	(Parker et al. 2007)

At the same, it was observed that the intermediate host plays a crucial role in transmitting the zoonotic virus from natural hosts to others. The intermediate host is an animal, and sometimes, domestic animals can be intermediate hosts. Several studies have observed that these intermediate hosts might suffer from the disease. For example, during the coronavirus transmission, swine acute diarrhea syndrome was noted when coronavirus (SARS-CoV and/or swine acute diarrhea syndrome coronavirus) was transmitted from bats to pigs (Chakraborty et al. 2020, Zhou et al. 2019). However, the intermediate hosts of the different CoVs are noted as pigs, civets, camels, cows, camelids, etc. The intermediate hosts for SARS-CoV-2 and monkeypox virus strain are not correctly understood yet and have not been specified.

Understanding the exact animal origin of any viral outbreak is not easy. However, it is very urgent and necessary to pinpoint the animal origin with the intermediate hosts of these two viruses (SARS-CoV-2 and monkeypox virus). With the shortage of data on the precise animal origin of any viral outbreak, future preventive measures to control the disease epidemics and pandemics cannot be done very effectively. Therefore, we propose multidisciplinary teamwork and collaborative approaches throughout the world among scientists involving (molecular) biologists, physicians, ecologists, veterinarians, virologists, health scientists, pathologists and computational biologists to quickly identify all animal sources of SARS-CoV-2 and monkeypox virus such as their main host, intermediate host, and animal reservoirs.

## Deadly viral outbreaks originated from wild animals

Viruses occur naturally, and over 320,000 different viruses can infect mammals. But, only about 200 of them can infect humans. Some of the previously reported deadly infectious diseases such as Ebola, Marburg, HIV-AIDS, Nipah, Hendra, and West Nile have originated from wild animals ranging from pangolins to primates and bats to civets (Wegner et al. 2022). Bats are the only true flying mammals, and they make up about one-quarter of all the known mammalian species in the world. Bats are also known to serve as natural reservoirs for the filoviruses, SARS-like coronaviruses, Nipah, and Hendra paramyxoviruses (Calisher et al. 2006). But, why bats attract more viral pathogens affecting humans than other animals is not clearly understood. Olival et al. tried to understand patterns of viral diversity in wildlife. In this direction, they have been attempting to understand the determinant that helps in the virus's spillover or cross-species transmission. They have illustrated the number of zoonotic viruses per species. These parameters were analyzed using the host taxonomy and human population within a species range and phylogenetic relatedness to humans. These factors might help to explain human-wildlife contact. The researchers have found that viral traits and phylogenetic host breadth are major prediction parameters of zoonotic potential (Olival et al. 2017).

The enduring COVID-19 pandemic has reportedly started from a wholesale animal market in Wuhan, the capital of China's Hubei province. The wet markets are famous throughout China. They sell various types of live wild animals that are often confined in small cages where the transfer of pathogens from animals to humans can quickly happen. In fact, the Severe Acute Respiratory Syndrome coronavirus or SARS-CoV-1 originated in China's Guangdong province in 2003. The outbreak was suspected of starting in a wet market. But, the exact wild animal origin of the virus has yet to be confirmed conclusively.

## The origin of ongoing pandemic virus SARS-CoV-2: is it still ambiguous animal origin?

The reports at the beginning have suggested bats be the potential reservoirs, and a paper, for example, by Zhou et al., showed 96% similarities at the whole-genome level with the bat coronavirus confirming SARS-CoV-2 originating from bats (Zhou et al. 2020). An article by Zhang et al. that appeared in Current Biology suggested pangolins to be the potential natural reservoir of the SARS-CoV-2-like coronaviruses (Zhang et al. 2020). In contrast, another paper by Liu et al. identified the pangolin coronavirus or pangolin-CoV-2020 to have genetic closeness to SARS-CoV-2. This study tried to understand the viral communities of Malayan pangolins (*Manis javanica*). However, the study did not show the COVID-19 emerging directly from the pangolin-CoV-2020. The study described that this animal (Malayan pangolins) might be one more host which might transmit the SARS-CoV-2 to humans (Liu et al. 2019). Lam et al. have illustrated pangolin-associated CoVs (coronaviruses) associated with two sub-lineages of SARS-CoV-2-related coronaviruses. One shows high similarity in the RBD (receptor-binding domain) to SARS-CoV-2. They have also concluded that pangolins might be a possible host. The animal should be removed from the wet market (Lam et al. 2020). Finally, some questions have been raised: First, is the pangolin the primary host of SARS-CoV-2 along with the bat? Second, does pangolin act as an intermediate host of SARS-CoV-2? Third, which one is the natural reservoir of SARS-CoV-2, bat or pangolin or both? However, future researchers should answer all those questions.

Additionally, another paper by Xiao et al. reported that the SARS-CoV-2 seemingly originated from recombination of the coronaviruses of the bat and the pangolin (Xiao et al. 2020). However, in addition to pangolins and bats, a recent paper by Dabravolski et al. has added the yak beta-coronavirus strain (YAK/HY24/CH/2017) to be the closest match of spike glycoproteins suggesting the SARS-CoV-2 originating from the yak as an intermediate host (Dabravolski and Kavalionak 2020). Rabalski et al. reported that the mink might be a host of SARS-CoV-2, and they observed mink to human jump of this virus (Rabalski et al. 2022). Griffin et al. reported that the North American deer mouse might be another host of SARS-CoV-2 (Griffin et al. 2021). Guo et al. reported that the hamsters are additional hosts of SARS-CoV-2 (Guo et al. 2022). Thus, it might create the ambiguities surrounding the animal origin of COVID-19 that continues to puzzle the scientific community at large. Several researchers have tried to unreveal the origin of the virus (Banerjee et al. 2021). Casadevall et al. also urge to finish the controversy of the origin of the SARS-CoV-2 virus (Casadevall et al. 2021).

## The zoonotic host of the recent transmitted zoonosis of monkeypox virus

Monkeypox is a zoonotic viral disease that re-emerges naturally. This disease occurs in densely forested regions of Africa, especially Central Africa and West Africa (Reynolds et al. 2019). It has been noted that monkey is the significant host of the monkeypox virus. Other than the monkey, several zoonotic hosts of the virus have been found, such as chimpanzees (Guagliardo et al. 2020), rodents (Salzer et al. 2013), prairie dogs (Weiner et al. 2019), etc. From serologic evidence, it was observed that this virus might transmit from rodents to individuals (Salzer et al. 2013). Weiner et al. have reported that the black-tailed prairie dogs might be one host of the virus (Weiner et al. 2019). In the USA, individuals, including children, were infected by prairie dogs. Rodents infected these prairie dogs. These animals were shipped from Ghana to the USA (Simpson et al. 2020). Similarly, understanding the zoonotic host of the virus is necessary, and such information can reduce the risk of zoonotic disease. Researchers noted several gaps: First, a better understanding of reservoirs, zoonotic hosts, and intermediate hosts. Second, it is needed to note the risks which are associated with the transmission of these zoonotic viral diseases. Therefore, knowledge is essential to comprehend the zoonotic host, the reservoir of the zoonotic virus, etc.

## Urgent need for long-term monitor to zoonosis of SARS-CoV-2 and monkeypox virus

The particular region, from where both the SARS-CoV-1 and SARS-CoV-2 have originated, harbors over 4.3 billion people as well, and they constitute over 60% of the world’s human population. The people are concentrated in 22% of the world’s total land area, leading to high density spreading across major cities and towns. Thus, the potential for transferring new viral diseases from wild animals to humans can effortlessly transpire due to increased human density. To make matters worse, the enduring climate change consequences and biodiversity loss may further aggregate future interspecies infectious disease transmissions. It is also necessary that the biodiversity loss consequences help in to increase the virus spillover frequency from the host. Therefore, health authorities in the region need to pay more attention to systematically monitor zoonotic disease transfer since it has the potential to create new disease outbreaks in the future.

Scientists who are studying the COVID-19's animal origin are divided into two distinct groups; one suspects the pangolins to be the transmitting source, while the other points to the bats. However, there could be three possibilities: the virus jumping directly from bats to humans or from pangolins to humans or from bats to other intermediate animal hosts like the domesticated wild yak as suggested above. Without performing detailed *in vitro* and *in vivo* studies supported by long-term zoonosis monitoring, concluding the exact origin of the virus cannot be scientifically ascertained. Therefore, we propose intensive scientific monitoring of zoonosis in the region to avoid repeating history with yet another pandemic originating from Asia's wet markets.

In summary, we would like to stress that creating an integrated multi-national Asia Pacific zoonosis monitoring task force with adequate funding from all countries in the region is necessary. Then only scientists can investigate the mechanisms involving the COVID-19's origin and transmission and future outbreaks from diverse wild animals in the area with a multidisciplinary scientific approach, which is lacking now. The region, by the way, holds about half (17 out of 36) of the world's biodiversity hotspots, but unsustainable human activities are threatening the survival of highly endangered fauna, flora, and fungi-associated unique ecosystems (Morand et al. 2014). Individual countries and their scientists alone cannot tackle the monitoring of zoonosis tasks in the region since it would be monumental. Until and unless all countries in the area start to work in partnerships to systematically monitor zoonosis through an integrated scientific team, the logical riddles to confirm the virus's origin of the SARS-CoV-1 or SARS-CoV-2 cannot be resolved timely and effectively.

Similarly, it is also necessary to systematically monitor the monkeypox virus's zoonotic course in those regions, especially the Democratic Republic of the Congo and associated regions such as West Africa and Central Africa, where the monkeypox virus is originated. At the same time, the virus spillover event and the frequency should be monitored on a long time basis and analyzed critically as well with the One Health concept be reinforced widely for tackling zoonotic viruses.

## Conclusion

The best ways for future preparedness for the zoonotic viruses and to stop the appearance and re-appearance of zoonosis need to understand the root cause of zoonosis, such as the natural reservoir of the virus, primary host, or host range, intermediate hosts, and the virus spillover events (spillover time, and frequency of infecting the one host to another or reservoir to human), etc. We should also prepare the research priorities of zoonosis, and strengthen drug and vaccine development. We have already proposed multidisciplinary teamwork among scientists to stop these two zoonotic viral diseases throughout the world. At the same time, it is also necessary to better understand lethal genes from whole-genome sequencing, genomic evolution, and phylogenetics, changing epidemiology patterns, and genomic diagnostics of these to viruses, especially monkeypox viruses. We urge policymakers to pay more attention in this direction to stop the emerging and reemerging zoonotic diseases.
